# MUC16/CA125 in the Context of Modular Proteins with an Annotated Role in Adhesion-Related Processes: *In Silico* Analysis

**DOI:** 10.3390/ijms130810387

**Published:** 2012-08-21

**Authors:** Miroslava Jankovic, Ninoslav Mitic

**Affiliations:** Department for Immunochemistry and Glycobiology, Institute for the Application of Nuclear Energy-INEP, University of Belgrade, Banatska 31 b, Belgrade 11080, Serbia; E-Mail: ninoslavm@inep.co.rs

**Keywords:** MUC16, CA125, *in silico*, conserved domain, adhesion, herpesvirus, yeast, sugar-binding, ion transport

## Abstract

Mucin 16 (MUC16) is a type I transmembrane protein, the extracellular portion of which is shed after proteolytic degradation and is denoted as CA125 antigen, a well known tumor marker for ovarian cancer. Regarding its polypeptide and glycan structures, as yet there is no detailed insight into their heterogeneity and ligand properties, which may greatly influence its function and biomarker potential. This study was aimed at obtaining further insight into the biological capacity of MUC16/CA125, using *in silico* analysis of corresponding mucin sequences, including similarity searches as well as GO (gene ontology)-based function prediction. The results obtained pointed to the similarities within extracellular serine/threonine rich regions of MUC16 to sequences of proteins expressed in evolutionary distant taxa, all having in common an annotated role in adhesion-related processes. Specifically, a homology to conserved domains from the family of herpesvirus major outer envelope protein (BLLF1) was found. In addition, the possible involvement of MUC16/CA125 in carbohydrate-binding interactions or cellular transport of protein/ion was suggested.

## 1. Introduction

Mucins comprise a family of secreted or transmembrane proteins, characterized by extensive *O*-glycosylation on multiple tandem repeats of proline/threonine/serine rich (PTS) amino acid sequences [1–4]. Owing to their structural specificities, mucins contribute to mucociliary defense, acting as physical and chemical barriers, and to innate immune defense as part of signal transduction pathways [5–9].

Mucin 16 (MUC16) is a type I transmembrane protein, the extracellular portion of which is shed after proteolytic degradation. This is denoted as CA125 antigen, a well known tumor marker for ovarian cancer [10–12]. It is placed in the mucin family according to the results of partial cloning of sequence, but due to its specific properties, such as *N*-glycan composition, MUC16 does not fit well into either class of mucin molecules [13–15]. It has an extremely long amino acid sequence, and the available data indicate that this is dominated by 56 SEA (sea urchin sperm protein, enterokinase, agrin) repeats and 2 ANK (ankyrin) repeats, which occur in diverse functionally different proteins [16]. SEA is an extracellular domain associated with *O*-glycosylation, which might regulate or assist binding to neighboring carbohydrate moieties [17]. The ankyrin repeats are tandemly repeated modules of about 33 amino acids, which are one of the most common protein–protein interaction motifs [18]. Regarding its polypeptide and glycan structures, as yet there is no detailed insight into their heterogeneity and ligand properties, which may greatly influence the function and biomarker potential of MUC16/CA125 [19–23].

This study was aimed at gaining more insight into the biological capacity of this mucin by exploiting a combination of computational and experimental approaches. Thus, we performed *in silico* analysis of corresponding mucin sequences, including similarity searches as well as GO (gene ontology)-based function prediction. Subsequently, selected computationally identified hits were experimentally validated based on CA125-immunoreactivity.

The results obtained pointed to similarities within extracellular serine/threonine (Ser/Thr) rich regions of Muc16 to protein sequences expressed in evolutionary distant taxa, as well as homology to conserved domains, all having in common an annotated role in adhesion-related processes.

## 2. Results and Discussion

Table 1 lists the highest scoring candidates (putative/uncharacterized protein hits not considered) obtained when the MUC16/CA125 sequence was submitted to BLAST similarity searches through the following protein databases: virus, bacteria, fungi, eukaryota.

The membrane glycoprotein (039781)/glycoprotein gp2 (Q6SV6W0) from *Equine herpesvirus* 1 as well as glycoprotein gp350-220 (E2GKY4) from *Epstein Barr virus* (EBV) were reported as viral hits exhibiting sequence similarity to the target sequence. Human and animal herpesviruses are large, enveloped virions with related glycoproteins incorporated into the virion envelope. Conservation is manifested at both the structural and functional level. Gp2 is a virion membrane protein involved in viral reproduction [24–26]. Gp350-220 is the most abundantly expressed part of the viral envelope and its binding to CD21 is an essential step in infection of B lymphocytes by the EBV [27–29].

Cell wall surface anchor family protein (B2ISC7/Q97P71) from *Streptococcus pneumoniae* and serine-rich adhesin for platelets (Q4L9P0) from *Staphylococcus haemolyticus* were reported as bacterial hits. GO annotation described these entries as having transmembrane transporter activity and virulence activity mediating binding to specific cells [30–33].

The search through the fungi database pointed to high scoring candidates, known to exhibit mucin-like properties: cell surface flocculin, Flo11 (E9P8M0) and Muc1p (C8ZAR8) from *Saccharomyces cerevisiae*. Ser/Thr rich regions in high scoring hits, are known to be involved in cell adhesion and pseudohyphal formation or binding to polysaccharides in the natural environment and/or efficient invasive growth on such substrates [34–39].

In addition, the search through the eukaryota database reported mucin-like proteophosphoglycan 5 (E9AEM9) from *Leishmania major* exhibiting similarity to MUC16. It belongs to a family of heterogeneous polypeptides of unusual composition and structure and is the major cell surface molecule of promastigotes known to mediate attachment to the vector [40]. In addition, it is able to activate complement, but is poorly immunogenic and behaves immunologically like a carbohydrate [41].

Taken together, the results obtained put MUC16 in the context of evolutionary distant modular proteins sharing common features in terms of GO functional categories: cellular component (GO:0005575), biological processes (GO:0008150), and molecular function (GO:0003674). Thus, the highest scoring reported candidates are associated with the membrane/cell wall/extracellular region and are involved in different types of adhesion processes based on protein-protein or protein-sugar binding.

All reported similarities were found within the extracellular Ser/Thr-rich regions of MUC16, which are typical of mucin molecules in general. No relation to annotated domains from available databases appeared, except for gp2/BLLF1 (herpesvirus major outer envelope glycoprotein) from conserved domain database (CDD) [42]. As already mentioned, BLLF1 (also termed gp 350/220) represents a major antigen responsible for production of neutralizing antibodies *in vivo*. Starting from these observations as well as reported data on elevated CA125 concentration in patients with different type of B cell lymphomas, which could be associated with EBV infection [43,44], anti-human CA125 antibodies were probed for reactivity with herpesviruses glycoproteins. Thus, EBV capsid antigen and HSV 1 antigen were probed with two classes of monoclonal antibodies to MUC16/CA125: OC125/OC125-like and M11/M11-like, reacting mainly with the repeated peptide sequences [13,14,45].

**Figure 1:**
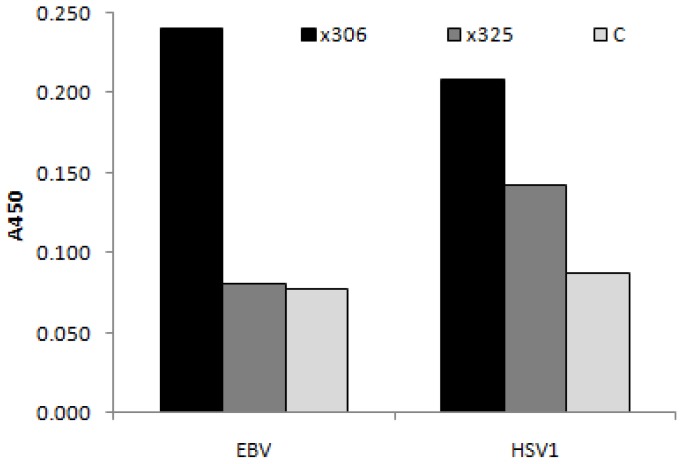
CA125-immunoreactivity of Herpesvirus antigens. Mouse monoclonal anti-human CA125 antibodies: clone X306 (OC125-like) and clone X325 (M-11 like) were allowed to react with immobilized Epstein-Barr Virus (EBV) capsid antigens or Herpes simplex virus type 1 antigens (HSV1). Binding was detected using biotinylated goat anti-mouse IgG and Vectastain Elite ABC reagent. The absorbance was measured at 450 nm. Non-specific binding was estimated using an irrelevant monoclonal anti-hCG antibody (c).

In a solid phase binding assay with immobilized targets (Figure 1), OC125-like antibody, but not M11-like antibody, gave a signal above background indicating measurable reactivity to EBV CA, but it was weak relative to the reaction with CA125. As for HSV 1 antigens, both antibodies gave measurable reactivity, being slightly higher for OC125-like antibody.

Generally, there is a phenomenon that unrelated organisms can have antigens in common [46–48]. Thus, it is well known that the agglutination test for EBV is based on the finding that it has an antigen in common with sheep and horse erythrocytes [43]. Moreover, fungal antigen crossreactivity is reported between *Candida* species and human ovarian carcinoma [49], whereas crossreaction of *Saccharomyces cerevisiae* was found in the human colon *i.e.*, in granulation tissue of inflamed colonic mucosa and peripheral leukocytes in patients with Crohns disease [50,51]. However, crude yeast extract, as a source of the identified mucin-like molecules, showed no trace of CA125-immunoreactivity (data not shown).

The available data indicate that 4% of 600 monoclonal antibodies against a large variety of viruses crossreacted with healthy host tissues and that heterologous immunity may be elicited even by very short common sequences (such as six amino acids) [52]. The biological meaning of such crossreactivity *i.e.*, heterologous immunity, in general, is not understood and also it is not known whether it may have any functional consequences.

As part of a strategy for assignment of structural/functional domains, a BLAST search starts with the basic assumption that higher sequence similarity increases confidence in function annotation transfer [53,54]. However, there is no threshold and homology does not always mean similar function. Thus, in addition to BLAST, protein function prediction software based on GO annotations were also used for computational analysis of CA125 sequence (Tables 2 and 3). Although, the reported matches had low probability scores, they put MUC16/CA125 in the context of modular proteins with an annotated role in adhesion-related processes. In terms molecular function, GO category: binding (GO:0005488) was associated with purine nucleotide (GO:0017076), metal ion/ion (GO:0046872/GO:0043197) or sugar binding (GO:0005529). The predicted sugar binding ability was related to 1,4-alpha-d-glucan (GO:0004339) and chitin (GO:0008061) [55,56]. In terms of biological processes, GO category: cellular process (GO:0009987) was associated with cell-matrix adhesion (GO:0007160), and GO category: physiological process (GO:0007582) was associated with cell growth (GO:0016049), transport (GO:0006810) and metabolism (GO:0008152). Thus, invasive growth (GO:0001403), cation transport (GO:0006812), *i.e.*, ATP synthesis coupled proton transport (GO:0015986) and polysaccharide metabolism (GO:0000272), were annotated, respectively [57,58].

So far, several lines of experimental evidence obtained on cancer- or pregnancy-associated MUC16/CA125 antigen, indicate possible involvement in adhesive/anti-adhesive processes during cancer progression or embryonic development [59–62]. The precise mechanisms of these processes are not fully explained. Generally, it is suggested that there is link between cell adhesion and ion transport. For instance, local extracellular pH levels at tumor focal adhesion sites modulate the strength of cell adhesion *i.e*., more protons leads to tighter adhesion and decreased migration [63]. These processes can involve different molecules, but there are data substantiating the existence of adhesion molecules with amino acid identity (40%) and immunologically cross-reactive to the beta subunit of Na/K-ATPase [64]. It is speculated that adhesive or anti-adhesive properties of a particular molecule may result from its influence on different transducing systems in the form of an ion pump, channel or carrier [64].

In addition, they can be dependent on its glycosylation status. It is known that mucins as ligands for cell-cell adhesion molecules (CAM) or as CAM themselves are an important part of the adhesion interaction network based on carbohydrate-binding interactions. Indeed, the results obtained indicated distinct GO terms, whose annotations, refer to lectin- or lectin-like interactions.

In terms of biological processes, besides cellular processes, carbohydrate-binding is also supposed to be relevant for physiological processes such as invasive growth (GO:0001403) or substrate-bound cell migration (GO:0006929). Thus, flocculin, identified as one of the high scoring hits, is associated with fimbrialike structures and it is involved in invasion and filamentous growth [65]. On the other hand, MUC16 was reported to be localized on the surface of uterodome (pinopode) protrusions of the endometrium, acting as a barrier for trophoblast adherence [62]. Cell-matrix contact structures, *i.e*., cellular protrusions can be morphologically different, but mechanisms of spreading are thought to be similar in normal and pathologically altered cells [66]. However, there are no data on flocculin or CA125 activities in terms of sugar-binding interactions.

MUC16 has a distinct evolutionary relationship with other transmembrane mucins. Using sequence comparison of well characterized mucin domains: SEA, NIDO, AMOP and VWD, it was shown that MUC16 evolved separately, before the divergence of birds and mammals [67]. Thus, in contrast to the others, it has homology in non-mammalian species, based on the SEA domain. In this study, the starting point was modular organization and the preposition that sharing evolutionary conserved structural and functional motifs, other than those already known, can give us more information about its position in the human interactome.

Collectively, the results obtained direct further investigation of CA125 antigen towards collecting data to substantiate the involvement of common conserved protein motifs in functional activities of evolutionarily diversified molecules, as has emerged from this study.

## 3. Experimental Section

### 3.1. Dataset

The protein sequence of human mucin 16 (MUC16) (ovarian cancer-related tumor marker CA125), accession number Q8WXI7 (UniProtKB, The Universal Protein Knowledgebase), accession number IPI00103552 (IPI, The International Protein Index), accession number NP_078966.2 (NCBI refSeq, National Center for Biotechnology Information Reference Sequence) was retrieved from public databases [68,69].

### 3.2. Similarity Search

The protein sequence of human mucin 16 (MUC16) was subjected to a similarity search in the Protein Knowledgebase (UniProtKB) using BLAST (Basic Local Alignment Search Tool) [70–72]. The following protein knowledgebases were searched: bacteria, viruses, fungi, eukaryota; and the highest scoring candidates were ranked under different parameter settings (threshold, matrix, filtering, gapped sequence).

### 3.3. Protein Function Prediction

Functions were assigned based on the homologues identified using protein function prediction servers: JAFA metaserver (Joined Assembly of Function Annotations) at http://jafa.burnham.org, PFP (Automated Protein Function Prediction) at http://dragon.bio.purdue.edu/pfp, and GO (GeneOntology) at http://www.geneontology.com, which gives a definition of functional context and provides machine-legible functional annotation [73–78].

### 3.4. CA125-Immunoreactivity

#### 3.4.1. Viral Antigens

Mouse monoclonal anti-human CA125 antibodies: clone X325 (M-11 like) and clone X306 (OC125-like) were from HyTest (PharmaCity, Turku, Finland). They were allowed to react with immobilized Epstein-Barr Virus (EBV) capsid antigens (CA), from Epstein-Barr Virus (EBV) VCA IgG kit (Virion/Serion GmbH, Wurzburg, Germany), or Herpes simplex virus type 1 (HSV 1) cell culture-derived antigens, from Herpes simplex virus type 1 IgG kit (Human GmbH, Wiesbaden, Germany). After incubation for 3 h at room temperature (RT), the wells were washed three times with 0.1 M PBS, pH 7.2 and biotinylated goat anti-mouse IgG (Vector Laboratories, Burlinghame, CA, USA) was added. Subsequent to incubation for 1 h, the wells were rinsed and Vectastain Elite ABC reagent (Vector Laboratories, Burlinghame, CA, USA) was added followed by incubation for 30 min. After another washing step, addition of TMB substrate solution and incubation for 10 min, the reaction was stopped with 0.16 M H_2_SO_4_. The absorbance was measured at 450 nm on a Wallac 1420 Multilabel Counter (Monza, Italy). In parallel, a control assay was performed with an irrelevant monoclonal anti-hCG IgG, clone 5008-SP-5 (Medix Biochemica, Kauniainen, Finland) to determine non-specific binding.

#### 3.4.2. Crude Yeast (*Saccharomyces cerevisiae*) Extract

Serial dilutions of crude yeast (*Saccharomyces cerevisiae*) extract in 0.01 M carbonate buffer, pH 9.2, were adsorbed on polystyrene test tubes (Spektar, Cacak, Serbia) overnight at 4 °C. The tubes were then rinsed three times with 0.1 M PBS, pH 7.2, blocked with 1% casein for 2 h at RT and rinsed again three times with 0.1 M PBS, pH 7.2. Reaction with the corresponding monoclonal anti-human CA125-antibodies was then allowed as described for viral antigens.

## 4. Conclusions

Since protein function has many facets and is highly contextual, bioinformatic data on the predicted GO molecular function of CA125 can be considered in the light of possible general principles shared across distant distinct, yet related proteins. The results obtained suggested a possible correlation between the role of the serine/threonine rich domain of yeast, acting as a sensor for extracellular osmotic pressure and that of the mucin domain of transmembrane mucins in monitoring extracellular ion gradients and pH [35,79,80]. In addition, a possible relationship has emerged between mucin participation in polarized growth and directional motility *i.e.*, amoeboid mechanisms of propulsion and mucin-like fungal proteins in pseudohyphal and filamentous growth involving sugar-substrate binding [81,82].

## Figures and Tables

**Table 1:** Sequence similarity search for Q8WXI7 (MUC16/CA125) entry.

Database	Hit *	Protein/*Conserved Domain* **	Organism	Length	Identity	Score	*E*-value
**Virus**	O39781/Q6SV6WO	MembraneGlycoprotein, gp2*/Gp2* **	*Equine herpesvirus 1*	806	25%	295	1 × 10^−23^
	E2GKY4	Glycoproteingp350-220*/BLLF1* **	*Epstein Barr virus*	877	26%	170	3 × 10^−9^
**Bacteria**	B2ISC7/Q97P71	Cell surface anchor family protein	*Streptococcus pneumoniae(strain GSP14)*	4695	17%	669	4 × 10^−66^
	Q4L9PO	Serine-rich adhesion for platelets	*Staphylococcus haemolyticus (strain JCSC1435)*	3608	20%	625	5 × 10^−61^
**Fungi**	E9P8M0	Cell surface flocculin	*Saccharomyces cerevisiae*	1630	24%	499	4 × 10^−47^
	C8ZAR8	Muc1p	*Saccharomyces cerevisiae*	1576	23%	429	6 × 10^−39^
**Eukaryota**	E9AEM9	Proteophosphoglycan 5	*Leishmania major*	17392	18%	1003	6 × 10^−105^

*E*-value: <1 × 10^−50^—homology; 0.01–1 × 10^−50^—may be homology; 0.01–10 not significant, distant homology; >10—random;

* searched by BLAST;

** search by CDD v2.26-38392 PSSMs.

**Table 2:** Predicted gene ontology (GO) categories for Q8WXI7 (MUC16/CA125) entry *.

Biological process			Molecular function			Cellular component		
GO	Score	Definition	GO	Score	Definition	GO	Score	Definition
0006812	5363	Cation transport	0005515	3567	Protein binding	0005624	2,4775	Membrane
0007155	4679	Cell adhesion	0004867	3371	Endopeptidase inhibitor activity	0005887	4,420	Integral to plasma membrane
0007275	4273	Development	0004872	3133	Receptor activity	0016020	4,305	Membrane
0006929	3310	Substrate-bound cell migration	0004672	2914	Protein kinase activity	0005622	3,129	Intracellular
0050652	2664	Polysachharide biosynthesis	0004674	2623	Ser/Thr kinase activity	0016021	2,363	Integral to membrane
0007166	2554	Cell surface receptor linked signal transduction	0005529	2445	Sugar binding	0005578	2,118	Extracellular matrix (sensu Metazoa)
0007165	2416	Signal transduction	0005524	2219	ATP binding	0005634	1,939	Nucleus
0006917	2213	Induction of apoptosis	0008270	2206	Zinc ion binding	0005856	1,764	Cytoskeleton
0008228	2079	Opsonization	0003804	2154	Coagulation factor Xa activity	0009897	1,633	External side of plasma membrane
0035162	2071	Embryonic hemopoiesis	0046703	2075	NK cell like-receptor binding	0005615	1,531	Extracellular space

* searched by http://dragon.bio.purdue.edu/pfp; selected results.

**Table 3:** Predicted GO categories for Q8WXI7 (MUC16/CA125) entry *.

Biological process			Molecular function			Cellular component		
GO/GO-Level	Score	Name	GO/GO-Level	Score	Name	GO/GO-Level	Score	Name
**0015986/6**	2.00	ATP synthesis coupled proton transport	0004339/6	2.00	Glucan 1,4-alpha glucosidase activity	0005886/3	1.00	Plasma membrane
**0006030/6**	2.00	Chitin metabolism	0005524/5	1.67	ATP binding	0016469/2	0.67	Proton-transporting two-sector ATPase complex
**0000272/6**	2.00	Polysaccharide catabolism	0008061/4	1.33	Chitin binding	0005615/2	0.67	Extracellular space
**0007160/4**	1.33	Cell matrix adhesion	0005515/2	0.67	Protein binding	-	-	-
**0007124/3**	1.00	Pseudohyphal growth	0005554/1	0.33	Molecular function unknown	-	-	-
**0001403/3**	1.00	Invasive growth (sensu *Saccharomyces*)	-	-	-	-	-	-

* searched by http://jafa.burnham.org; selected results.
